# RASSF1A inhibits PDGFB-driven malignant phenotypes of nasopharyngeal carcinoma cells in a YAP1-dependent manner

**DOI:** 10.1038/s41419-020-03054-z

**Published:** 2020-10-14

**Authors:** Ying-Ying Liang, Xu-Bin Deng, Xian-Tao Lin, Li-Li Jiang, Xiao-Ting Huang, Zhi-Wen Mo, Ya-Wei Yuan, Muy-Teck Teh

**Affiliations:** 1 Department of Radiation Oncology, Affiliated Cancer Hospital & Institute of Guangzhou Medical University, Guangzhou, China; 2 Department of Internal Oncology, Affiliated Cancer Hospital & Institute of Guangzhou Medical University, Guangzhou, China; 3 Department of Internal Oncology, The First Affiliated Hospital of Hainan Medical University, Haiko, China; 4 Guangzhou Municipal and Guangdong Provincial Key Laboratory of Protein Modification and Degradation, School of Basic Medical Science, Guangzhou Medical University, Guangzhou, China; 5 Cancer Research Institute, Affiliated Cancer Hospital & Institute of Guangzhou Medical University, Guangzhou, China; 6 Centre for Oral Immunobiology and Regenerative Medicine, Institute of Dentistry, Barts & The London School of Medicine and Dentistry, Queen Mary University of London, London, England United Kingdom; 7 China-British Joint Molecular Head and Neck Cancer Research Laboratory, Affiliated Stomatological Hospital of Guizhou Medical University, Guizhou, China

**Keywords:** Cancer stem cells, Head and neck cancer, Tumour biomarkers, Cell signalling

## Abstract

Nasopharyngeal carcinoma (NPC) is a highly aggressive tumor characterized by distant metastasis. Deletion or down-regulation of the tumor suppressor protein ras-association domain family protein1 isoform A (*RASSF1A*) has been confirmed to be a key event in NPC progression; however, little is known about the effects or underlying mechanism of RASSF1A on the malignant phenotype. In the present study, we observed that RASSF1A expression inhibited the malignant phenotypes of NPC cells. Stable silencing of RASSF1A in NPC cell lines induced self-renewal properties and tumorigenicity in vivo/in vitro and the acquisition of an invasive phenotype in vitro. Mechanistically, RASSF1A inactivated Yes-associated Protein 1 (YAP1), a transcriptional coactivator, through actin remodeling, which further contributed to Platelet Derived Growth Factor Subunit B (*PDGFB*) transcription inhibition. Treatment with ectopic PDGFB partially increased the malignancy of NPC cells with transient knockdown of YAP1. Collectively, these findings suggest that RASSF1A inhibits malignant phenotypes by repressing PDGFB expression in a YAP1-dependent manner. PDGFB may serve as a potential interest of therapeutic regulators in patients with metastatic NPC.

## Introduction

Ras-association domain family protein1 isoform A (*RASSF1A*) is a well-known tumor suppressor protein inactivated by a combination of genetic and epigenetic mechanisms in various human cancers. *RASSF1A* is located on chromosome 3p21.3 and is downregulated in human tumor cells most frequently by promoter methylation and infrequently by mutation or deletion^1^. *RASSF1A* contains a ras-association domain, an ataxia telangiectasia mutant (ATM) kinase phosphorylation site, and Sav-RASSF-Hpo (SARAH) protein interaction domain in C-terminus, and its N-terminal sequence contains a diacyl glycerol binding domain^2–4^. RASSF1A lacks obvious enzymatic activity but may serve as a scaffold protein for signaling complexes by binding to key signaling mediators. The SARAH domain is a key feature of Hippo signaling pathway components, and the interaction of Sav, Rassf, and Hippo is accomplished *via* this domain. The Hippo pathway is a kinase cascade connecting the tumor suppressor Hippo (Mst1 and Mst2 in mammals) to the Yki protein (YAP1 (Yes-associated protein 1) and TAZ (Tafazzin) in mammals), a transcriptional coactivator of target genes involved in cell proliferation, cell cycle regulation, and apoptosis^5,6^. RASSF1A interacts with Mst1/2 *via* its SARAH domain and promotes the formation of an inhibitory complex comprising RAF1 and MST1/2, which then inhibits Lats1 phosphorylation and retains inactivated YAP1 in the cytoplasm^7^. It was also demonstrated that RASSF1A positively regulating Mst1 apoptotic activity, further leading to histone H2B phosphorylation, a hallmark of chromatin condensation^8^. The key upstream repressor of YAP1/TAZ activation is the Hippo (MST1/2-LATS1/2) pathway and apart from it, YAP1/TAZ could be mechanically activated by Integrin, PI3K-AKT and G-protein coupled receptor signals, all of which antagonize the Hippo pathway^9^. Accumulating studies have reported that RASSF1A triggers tyrosine phosphorylation of YAP1 and modulates its activation during various processes, including injury, inflammation, and carcinogenesis^10^. Overexpression of RASSF1A significantly inhibits cell proliferation and induces apoptosis by inhibiting the oncogenic functions of YAP1^11^. Acting as a downstream effector of the Hippo pathway, YAP1 has been identified as a proto-oncogene, as it acts by binding to the transcription factor TEAD1–4 (TEA domain family member, the major partner of YAP1 in its function in the Hippo pathway) and subsequently activates the transcription of genes involved in cell survival/proliferation and suppresses the transcription of apoptotic genes such as *c-Myc*, *OCT4, CYR61*, and *CTGF*^12–14^.

Nasopharyngeal carcinoma (NPC) is one of the most common malignancies in South China and Southeast Asia. NPC has the highest metastasis rate among head and neck cancers, and patients with distant metastasis experience higher rates of treatment failure^15,16^. Cancer stem cells (CSCs) are a small subpopulation of cells residing in tumors. CSCs of NPC have self-renewal, differentiation, and tumorigenic capabilities and are considered the cause of therapeutic resistance, tumor recurrence, and metastasis^17–19^. Variable expression of HIPPO-TAZ regulated by cisplatin treatment^20^ or by EBV-LMP1^21^ in NPC cells contributes to cancer stem cell-like properties and epithelial-mesenchymal transition. A high frequency of *RASSF1A* inactivation or down-regulation by gene promoter hypermethylation has been observed in NPC^22,23^. RASSF1A impairs cell proliferation in vitro and in vivo^24^, and methylation of its promoter has been linked to unfavorable prognosis in patients with NPC^25,26^. Restoration of RASSF1A expression is difficult due to technical issues and is accompanied by unpredictable complications; thus, determining its downstream effectors is necessary.

In the present study, we demonstrated that RASSF1A impairs malignant phenotypes by inhibiting YAP1-mediated expression of PDGFB during multiple steps of NPC carcinogenesis.

## Materials and methods

### Cell culture, reagents and ELISAs

Well-differentiated CNE-1, poorly differentiated CNE-2 and SUNE-1 are commonly used NPC cell lines in scientific research. All of them were maintained in DMEM (Invitrogen, USA) supplemented with 10% fetal bovine serum (FBS; Invitrogen, USA) at 37 °C and 5% CO_2_. Cells were plated in 6-well plates (Corning, USA) and treated with humane recombinant PDGF-BB (220-BB-010, R&D Systems, USA) or Immunoglobulin G (IgG) control (AB-108-C, R&D Systems, USA) or neutralizing antibody against PDGF-BB (AB-220-NA, R&D Systems, USA) or latrunculin b (LTB, ab144291, Abcam, UK) 12 h after plating. The PDGF-BB level in supernatant of cultured cell was measured using ELISA Kits for PDGF-BB (DBB00, R&D Systems, USA) according to the manufacturer’s instructions.

### Cell proliferation assay and spheroid formation assay

1 × 10^3^ cells suspended in 200 μl of medium were seeded into a 96-well plate (Corning, USA) and cultured under normal conditions. At various time points after seeding, the cells in each well were stained with MTS (G5421, Promega, USA), and the OD490 was determined with a microplate reader. Single-cell suspensions containing 800–1000 cells were seeded in 12-well ultra-low-attachment culture plates (Corning, USA) and cultured in serum-free DMEM/F12 (11320082, Invitrogen, USA)supplemented with 20 ng/ml EGF (PHG0311, Invitrogen, USA) and 10 ng/ml bFGF (PHG0360, Invitrogen, USA) for 10–14 days. The formed spheroids were counted and representative images were acquired *via* microscopy.

### Plasmid construction and transfection

A *RASSF1* expression construct was generated by subcloning PCR amplified full-length human *RASSF1* (transcript variant A) cDNA into a plasmid. Cells stably expressing either RASSF1A short hairpin RNA (shRNA) targeting *RASSF1* (transcript variant A) or a scrambled, non targeting shRNA were generated using the LV3 plasmid according to the manufacturer’s instructions. The target sequences of RASSF1A shRNA-2 and shRNA-5 were 5′-CGTGGACGAGCCTGTGGAG-3′ and 5′-GCTGAGATTGAGCAGAAGA-3′, respectively. Retroviral production and infection were performed as previously described^27^, and stable cell lines were selected using 1–3 mg/ml puromycin for 5–7 days.

### Small interfering RNA (siRNA) transfection

The siRNA mixed sequences targeting YAP1 (L-012200–00–0005) and PDGFB (L-011749–00–0005) were purchased from Dharmacon (USA). A non-targeting siRNA sequence (D-001210–01–05, Dharmacon, USA) was used as negative control. Cells (2 × 10^5^ cells per well) were seeded in a 6-well tissue culture dish, and the siRNAs (50 nM) were added 24 h later using RNAiMAX reagent (13778–075, Invitrogen, USA). The transfected cells were incubated for 6 h and were then supplied with fresh medium containing serum.

### Transwell assay

Migration and invasion assays were performed using cell culture inserts with transparent polyethylene terephthalate filters with an 8 μm pore size (354480, Corning, USA) with (for invasion assays) or without (for migration assays) matrigel coating. CNE-2 cells (2 × 10^4^) and CNE-1 cells (5 × 10^4^) suspended in 200 μl of serum-free DMEM with or without 12 h pretreatment with mitomycin C (1 ug/ml), then were added to the upper chambers, and 800 μl of DMEM containing 10% FBS was added to the bottom chambers. After incubation for 20 h at 37 °C, the cells on the upper filter were removed, and the cells that invaded the membrane or migrated to its lower surface were fixed with methanol and stained with crystal violet. Three optical fields were randomly selected from each of three inserts to calculate the average numbers of migrated or invaded cells.

### Immunofluorescence analysis

Cells were blocked for 30 min in 5% BSA and incubated with phalloidin (A12379, Invitrogen, USA) at a 1:100 dilution for 1 h in the dark at room temperature. Then, slides were stained with DAPI (D1306, Invitrogen, USA) for 5 min to visualize nuclei. Images were acquired *via* high-throughput confocal microscopy.

### Western blot analysis

Nuclear and cytosolic fractionation was performed using a subcellular protein fractionation kit (78840, Invitrogen, USA). Immunoblotting was performed according to standard methods as described previously^28^. Primary antibodies against the following proteins were used at a concentration of 1:1000: RASSF1A (ab97749) from Abcam (UK) and YAP1 (#14074), α-tubulin (#3873), Histone-H3 (#4499), E-cadherin (#3195), Vimentin (#5741), β-actin (#4970), and GAPDH (#2118) from Cell Signaling Technology (USA).

### Real-time reverse transcription-quantitative PCR (qRT-PCR)

The mRNA levels of *YAP1*, *PDGFB*, Cysteine-rich angiogenic inducer 61 (*CYR61*) and connective tissue growth factor (*CTGF*) were measured by real-time qRT-PCR according to the manufacturer’s instructions^27^. The house keeping gene *GAPDH* was used as the internal normalization control to calculate the mRNA levels of the different genes.

### In vivo tumorigenicity experiments

The protocol for the xenograft experiments was approved by the Institutional Animal Care and Use Committee of Sun Yat-Sen University Cancer Center. Female BALB/c nude mice (4–6 weeks old, 15–18 g; Animal Center of Guangdong Province) were housed in barrier facilities. Mice were randomly divided into groups. The indicated tumor cells (5 × 10^4^ or 2 × 10^5^) were suspended in 100 μl of sterile PBS containing 50% matrigel (356243, BD Biosciences, USA) and were subcutaneously inoculated into the mice (*n* = 10 per group). The mice were monitored daily for palpable tumor formation. All mice were euthanized 5 weeks after injection. The tumor-initiating cell frequency (TIF) was calculated using extreme limiting dilution analysis (ELDA) software (http://bioinf.wehi.edu.au/software/elda/).

### Genome-wide expression profiling, pathway analysis

Genome-wide expression profiling of RASSF1A-overexpressing CNE-2 and RASSF1A-depleted CNE-1 cells and their corresponding control vector cells was performed by Sagene Biotech Co. (Guangzhou, China). Raw data were normalized by log2 transformation and *z*-score calculation [(individual log transformed signal intensity (LS) value- mean of all LS values)/S.D. of all LS values]. Normalized data were used for statistical analyses.

### Statistics

All statistical analyses were carried out using SPSS 19.0 (IBM, Chicago, IL, USA). The data are presented as the means ± standard errors (SEs) of at least three independent experiments. Two-tailed Student’s *t* test was used to compare data between two groups. For all cell culture experiments, technical triplicates were evaluated in at least three independent experiments. *P* values of <0.05 were considered statistically significant.

## Results

### RASSF1A suppresses self-renewal properties and tumorigenicities of NPC cells

**Fig. 1:**
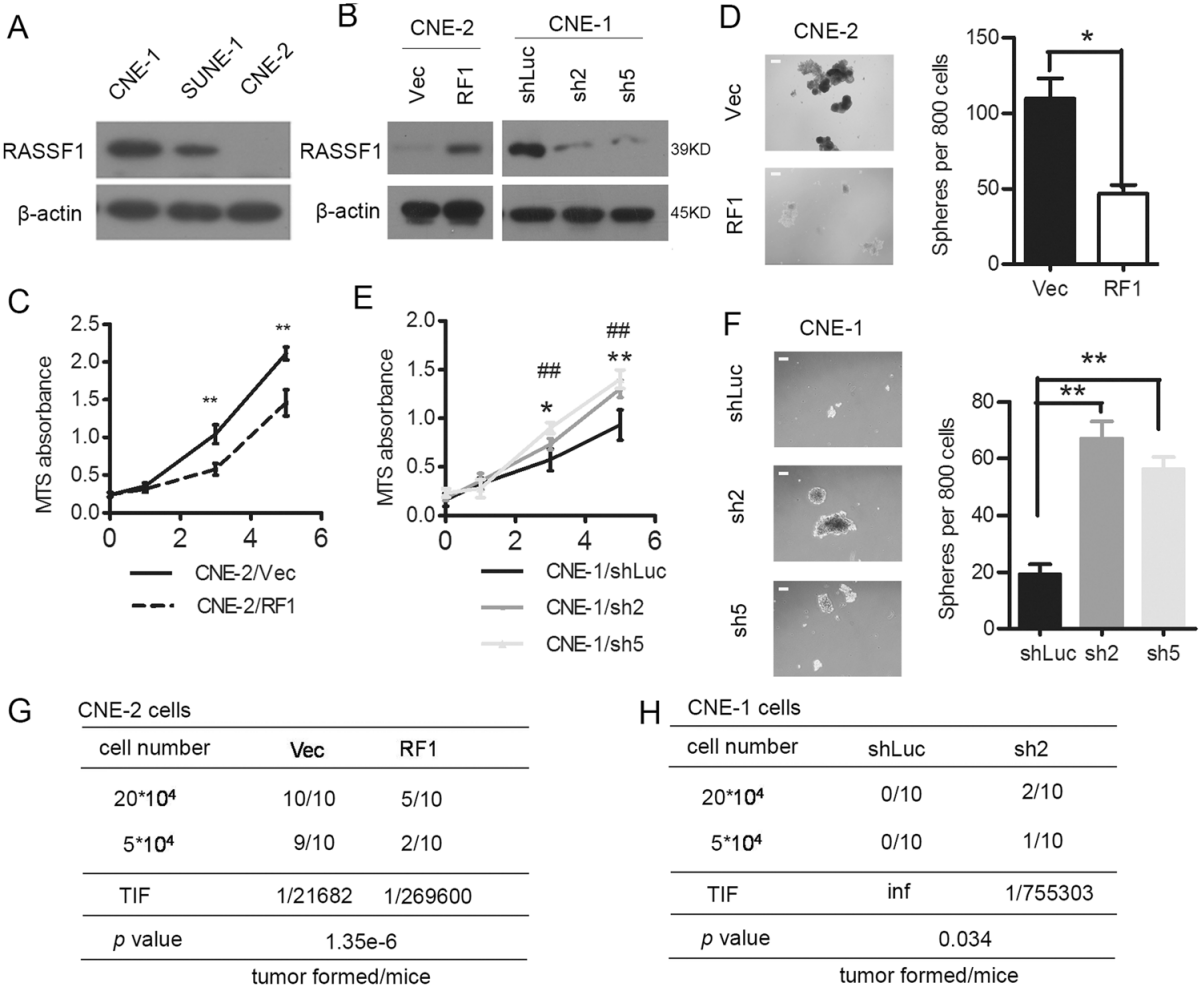
Expression of RASSF1A correlates with the self-renewal properties and tumorigenicity of NPC cells. **a** Protein expression levels of RASSF1A in NPC cells; β-actin was used as the loading control. **b**–**d** CNE-2 cells stably transfected with overexpressing RASSF1A (RF1) or with empty vector (Vec) were analyzedas follows. (**b**, left panel) RASSF1A protein expression levels were determined by western blot analysis; β-actin was used as the loading control. **c** A cell proliferation curve was constructed from MTS assay results, the data are presented as the mean ± S.D. values, **p < 0.01, Student’s *t* test. **d** Single-cell suspensions were seeded in ultra-low-attachment culture plates. The formed spheroids were counted *via* microscopy, and representative images are shown. The representative images and numbers of RASSF1A-overexpressing and control cells were compared, *p < 0.05, Student’s *t* test. Scale bar: 200 µm. **b**, **e**, **f** CNE-1 cells stably transfected with shRNA targeting RASSF1A (sh2, sh5) or scrambled shRNA (shLuc) were analyzedas follows. (**b**, right panel) Western blot analysis of RASSF1A expression; β-actin was used as the loading control. **e** A cell proliferation curve was constructed from MTS assay results, the data are presented as the mean ± S.D. values, *p < 0.05 and **p < 0.01 for CNE-1/sh2 cells compared with CNE-1/shLuc cells; ^##^*p* < 0.01 for CNE-1/sh5 cells compared with CNE-1/shLuc cells; Student’s *t* test. **f** Single-cell suspensions were seeded in ultra-low-attachment culture plates. The formed spheroids were counted via microscopy, and representative images of CNE-1 cells transfected with RASSF1A shRNAs or negative control scrambled shRNA are shown. **g** A total of 2 × 10^5^ (upper) and 5 × 10^4^ (lower) RASSF1A-overexpressing and its control CNE-2 cells were subcutaneously injected into NOD/SCID mice (*n* = 10 mice/group). Summary of tumorigenicity in mice were shown. The TIF and *p* value were calculated using ELDA software. **h** A total of 2 × 10^5^ (upper) and 5 × 10^4^ (lower) RASSF1A shRNA- or scrambled shRNA-targeting CNE-1 cells were subcutaneously injected into NOD/SCID mice (*n* = 10 mice/group). Summary of tumorigenicity in mice were shown. The TIF and *p* value were calculated using ELDA software.

We evaluated RASSF1A expression in commonly used NPC cell lines and found that CNE-1 cells, which exhibits a well differentiated phenotype, had higher levels of RASSF1A, whereas poor-differentiated CNE-2 had lowest level of RASSF1A (Fig. 1a). To determine whether RASSF1A plays oncogenic roles in NPC cells, we infected CNE-2 NPC cells with wild-type RASSF1A- or empty control vector-expressing lentivirus (Fig. 1b, left panel). Significantly fewer CNE-2 cells transfected with RASSF1A were observed in the proliferation assay (Fig. 1c). In addition, the RASSF1A-overexpressing CNE-2 NPC cells formed smaller and fewer spheres than the empty vector-transfected cells (Fig. 1d). To determine whether NPC cells with RASSF1A overexpression can exhibit tumorigenicity in vivo, we injected CNE-2 cells with RASSF1A- or empty vector-expressing lentivirus at different inoculation densities into nude mice. The TIF significantly reduced in RASSF1A-overexpressing CNE2 cells compared with control cells (Figs. 1g and S1).

We subsequently studied whether depletion of RASSF1A is sufficient to confer self-renewal properties. CNE-1 cell lines with high levels of RASSF1A expression were selected for loss-of-function assays. We knocked down RASSF1A in CNE-1 NPC cells and confirmed the reduced expression of RASSF1A by western blot analysis (Fig. 1b, right panel). RASSF1A knockdown increased the number of viable CNE-1 cells (Fig. 1e) and their anchorage independent growth, as these cells formed more and larger spheres than those transduced with scrambled shRNA (Fig. 1f). To rule out the off target effect of shRNA, we re-expressed RASSF1A in RASSF1A-knockdown CNE-1 cells with silent mutation in shRNA target region for conferring resistance. Restoration of RASSF1A significantly reduced the accelerated ability of proliferation and sphere formation leaded by RASSF1A-knockdown in CNE-1 cells (Fig. S2A–C). ShRNA targeting region also matched other transcript variant of RASSF1. RASSF1D isoform was at undetectable mRNA level in CNE-1 cells (data not shown), we therefore re-expressed RASSF1B in CNE-1/sh2 cells and re-expressed shRNA-resistant RASSF1C in CEN-1/sh5 cells to mimic the intrinsic expression level. After period of 1 week’s observation, we did not find significant changed of phenotypes (Fig. S2D–F), which was inconsistent with literature reported that RASSF1C plays oncogenic function. It may probably due to CNE-1 cells had relatively low intrinsic expression of RASSF1C. Next we injected shRNA-mediated RASSF1A-knockdown CNE-1 cells and control cells at different inoculation densities into NOD/SCID mice, control CNE-1 cells failed to form tumors in nude mice after the full observation period. However, the RASSF1A-silenced CEN-1 cells showed increased TIF (Figs. 1h and S1).

### RASSF1A regulated expression of markers involved in cell movement and ultimately inhibits the invasive phenotype of NPC cells

**Fig. 2:**
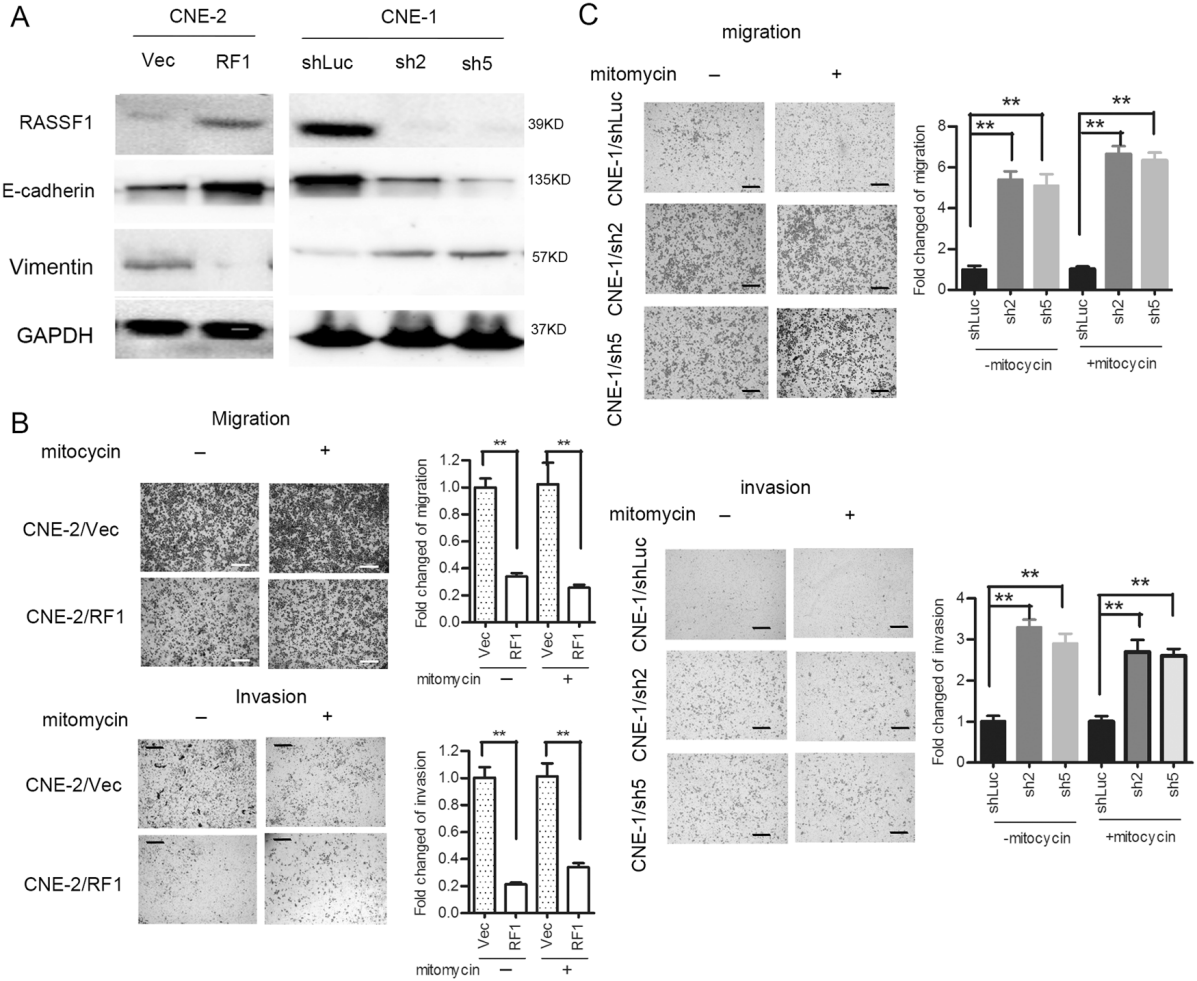
RASSF1A regulated expression of molecules involved in cell movement and ultimately inhibits the motility and invasive phenotype of NPC cells. **a** Western blot analysis of E-cadherin and vimentin in RASSF1A-overexpressing (left panel) CNE-2 cells, RASSF1A-depleted (right panel) CNE-1 cells and their corresponding control cells, GAPDH was used as the loading control. **b** RASSF1A markedly attenuated the migration and invasion characteristics of CNE-2 cells with or without presence of mitomycin C(1 ug/ml). Representative images of the migration assay (upper) and invasion assay (lower) of RASSF1A-overexpressing and control CNE-2 cells. The data are presented as the mean ± S.D. values, **p < 0.01, Student’s *t* test. Scale bar: 100 µm. **c** RASSF1A depletion enhanced CNE-1 cell migration and invasion with or without presence of mitomycin C (1 ug/ml), as determined by migration (upper pannels) and invasion (lower pannels) assays, the data are presented as the mean ± S.D. values, **p < 0.01, Student’s *t* test. Scale bar: 100 µm.

The above results suggest that RASSF1A depletion induces a CSC characteristic. CSCs are responsible for increased motility and the invasive phenotype of cancer cells. Thus, we evaluated the expression of molecules involved in the process of cell-cell adhesion and cell movement in NPC cell lines^29,30^. RASSF1A-overexpressing CNE-2 cells showed increased E-cadherin expression and decreased vimentin expression. In contrast, RASSF1A-depleted CNE-1 cells had undergone EMT, as indicated by the concomitantly decreased E-cadherin expression and increased vimentin expression (Fig. 2a). Next, we performed cell invasion and migration assays using Transwell chambers coated with or without an extracellular matrix. Overexpression of RASSF1A significantly reduced the ability of CNE-2 cells to migrate in the transwell chamber and invade the extracellular matrix layer. To rule out the influence of cell proliferation, we repeated the assay of migration and invasion in the presence of mitomycin C, an anti-proliferative agent, and observed the significant change of migrated and invaded ability of indicated cells (Fig. 2b). We then performed the same experiment and observed the opposite result in CNE-1 cells with stable knockdown of RASSF1A (Fig. 2c). The above data suggested RASSF1Acould inhibit the motility and invasive phenotype of NPC cells.

### PDGFB induction *via* RASSF1A deletion is required for the malignant phenotypes of NPC cells

**Fig. 3:**
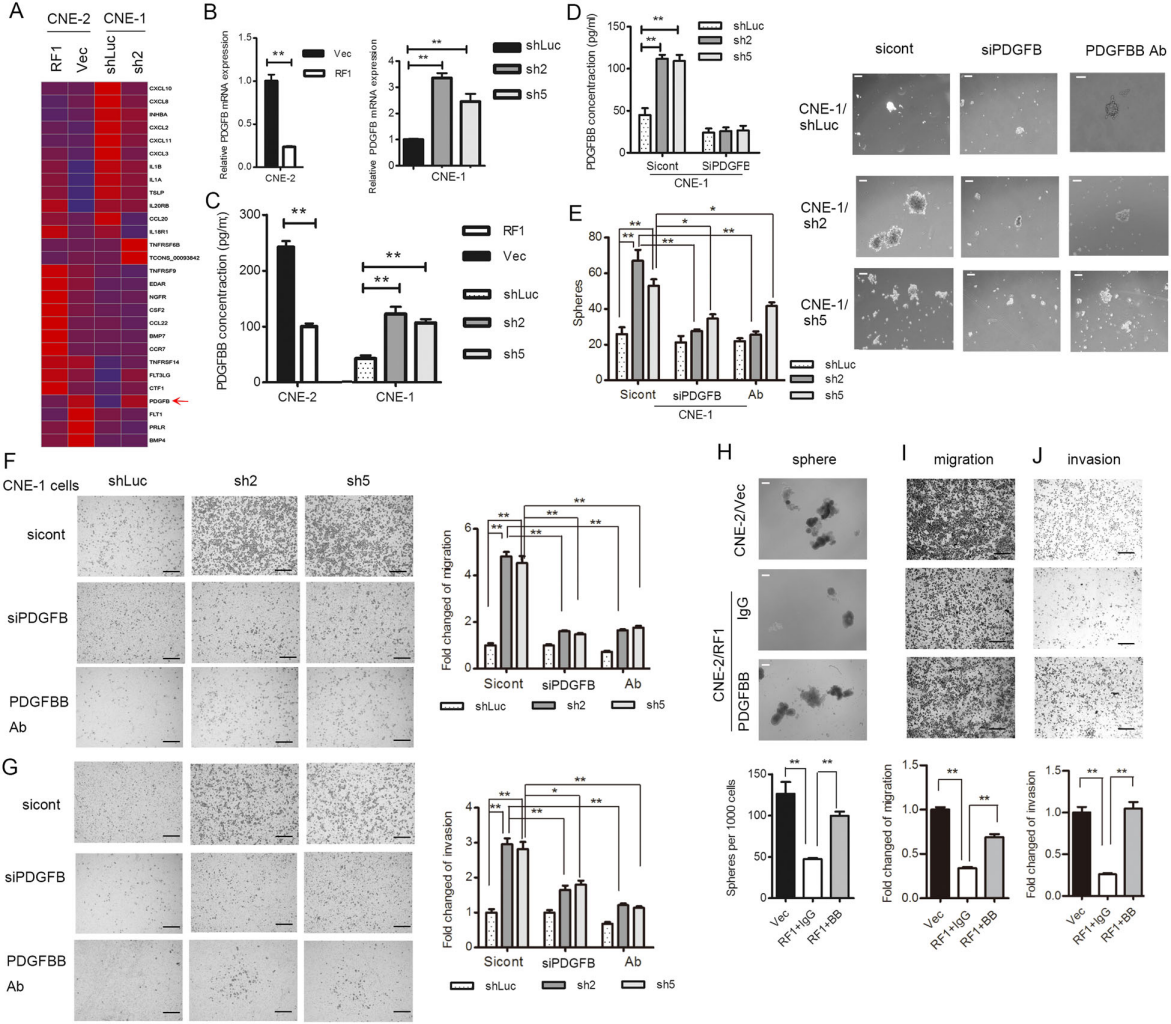
PDGFB is crucial for maintaining malignant properties induced by RASSF1A in NPC cells. **a** A heat map generated using the significantly changed genes categorized in the “cytokine-cytokine receptor interaction pathway” is shown. **b**, **c** mRNA expression (**b**) was evaluated by qRT-PCR and protein concentration by ELISA (**c**) in CM of RASSF1A-overexpressing CNE-2 cells, RASSF1A-depleted CNE-1 cells and their corresponding control cells, The data are presented as the mean ± S.D. values, **p < 0.01, Student’s *t* test. **d**–**g** PDGFB was transiently knocked down with a pool of siRNA or treated with a neutralizing antibody for PDGF-BB (10 µg/mL) in RASSF1A-depleted CNE-1 cells. PDGF-BB secretion in the CM was measured by ELISA (**d**), **p < 0.01, Student’s *t* test. **e** Number of spheroids formed was determined via microscopy, and representative images (**e** left panel) are shown. The formed spheroids were compared (**e** right panel), the data are presented as the mean ± S.D. values, *p < 0.05, **p < 0.01, Student’s *t* test; ns: non-sinificant. Scale bar: 200 µm. Representative images of the migration assay (**f**) and invasion assay (**g**) are shown, the data are presented as the mean ± S.D. values, **p < 0.01, Student’s *t* test. Scale bar: 100 µm. **h**–**j** Recombinant PDGF-BB or IgG was added to RASSF1A-overexpressing CNE-2 cells. Representative images of sphere formation (**h**) (Scale bar: 200 µm.), migration (**i**) and invasion (**j**) assays (Scale bar: 100 µm) of RASSF1A-overexpressing CNE-2 cells treated with PDGF-BB (the culture medium was supplemented with 20 ng/ml or an equal volume of control IgG) are shown, The data are presented as the mean ± S.D. values, *p < 0.05, **p < 0.01, Student’s *t* test.

To reveal the underlying mechanisms by which RASSF1A modulates the stem-like characteristics of NPC cells, we performed gene expression profiling to compare gene transcription in RASSF1A-overexpressing, RASSF1A-silenced and control NPC cells. Gene set enrichment analysis was used to identify a significant association between gene sets changed by modulation of RASSF1A expression. Then, Kyoto Encyclopedia of Genes and Genomes pathway analysis was applied to identify key pathways involved in this process. In NPC cells, RASSF1A was most significantly involved in the regulation of genes that encode cytokines (Fig. S3). We used a venn diagram to compare the differentially expressed cytokines and found that *PDGFB* expression was significantly changed in both NPC cell lines (Fig. 3a). We then confirmed by using qRT-PCR that the mRNA level of *PDGFB* was significantly lower in RASSF1A-overexpressing cells than in empty vector-expressing (control) cells, whereas the mRNA levels of *PDGFB* were 2.5–3.5-fold higher in RASSF1A-depleted CNE-1 cells than in control cells (Fig. 3b). PDGFB is released into the microenvironment as a homodimer (PDGF-BB). To confirm that the changes in *PDGFB* mRNA expression affected its protein level, we measured the amount of PDGF-BB secretion in the supernatant of cultured cells. The PDGF-BB concentration was significantly lower in the supernatant of RASSF1A-overexpressing cells than in that of their corresponding control cells. In contrast, the PDGF-BB concentration was higher in the supernatant of RASSF1A- depleted CNE-1 cells than in that of control cells (Fig. 3c).

We next sought to determine whether PDGF-BB secretion controls the acquirement of malignant phenotypes of NPC cells. *PDGFB* was transiently silenced in RASSF1A-depleted CNE-1 cells (Fig. 3d). The RASSF1A silencing-induced increase in the number of spheres formed was partially reduced by *PDGFB* knockdown or by neutralizing antibody against PDGF-BB treatment (Fig. 3e). Moreover, *PDGFB* knockdown or neutralizing antibody treatment suppressed the migration and invasion of RASSF1A-depleted CNE-1 cells (Fig. 3f, g).

Furthermore, addition of PDGF-BB reversed the malignant phenotypes, including sphere formation, migration and invasion, of RASSF1A-overexpressing CNE-2 cells (Fig. 3h, j). Collectively, these data suggest that the modulation of malignant NPC cell phenotypes by RASSF1A is dependent on PDGFB signaling.

### RASSF1A regulates actin cytoskeletal rearrangement and YAP1 activation

**Fig. 4:**
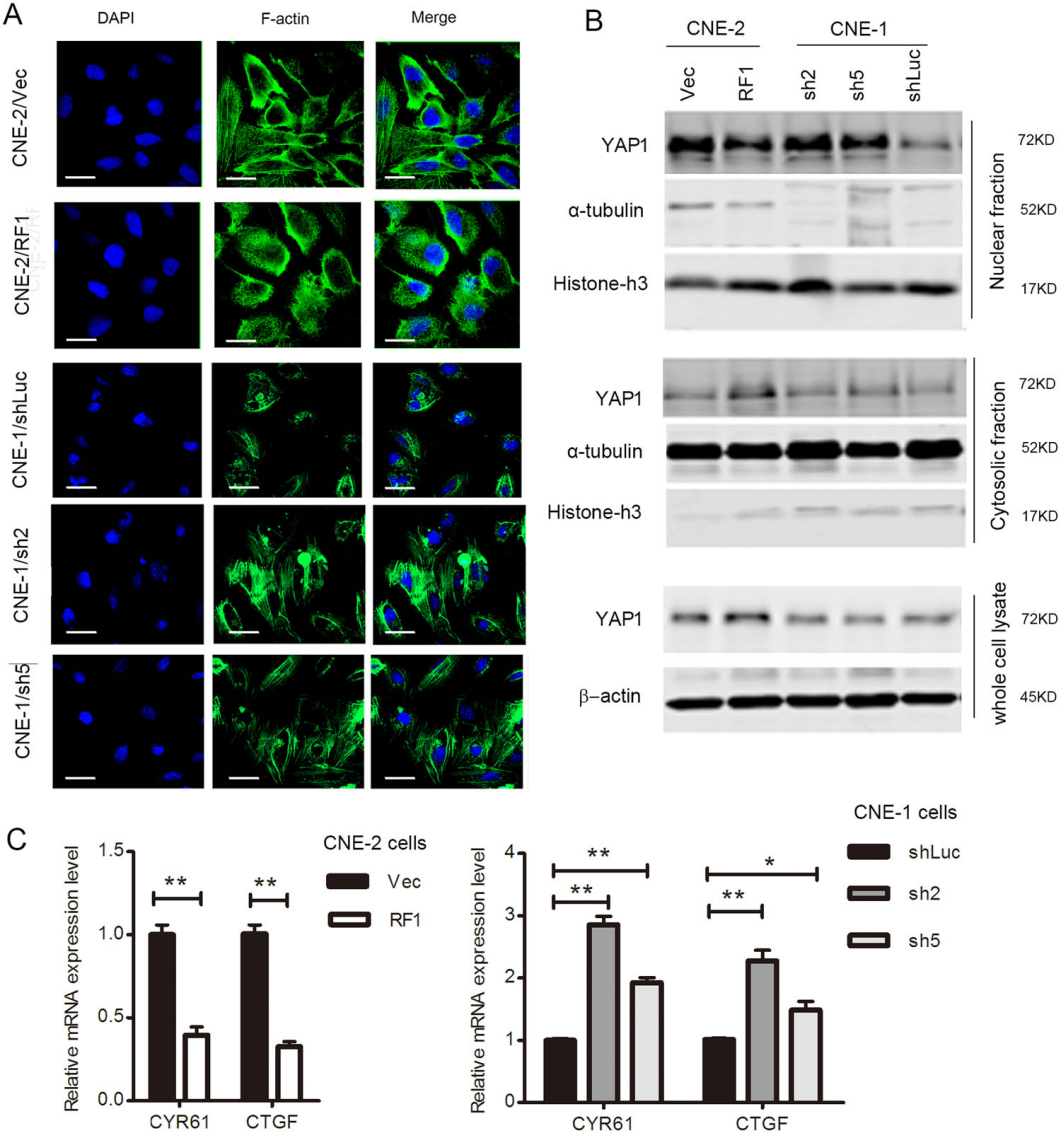
RASSF1A induces actin cytoskeletal rearrangement and inhibits YAP1 activation. **a** Representative images of F-actin stained with phalloidin and observed by immunofluorescence microscopy. Nuclei were visualized by DAPI staining, scale bar: 25 µm. **b** YAP1 expression in the total, cytosolic or nuclear fractions of RASSF1A-overexpressing CNE-2 cells, RASSF1A-delepted CNE-1 cells and their corresponding control cells were determined by western blot analysis. **c** mRNA quantification of *CYR61* and *CTGF* by using qRT-PCR; *GAPDH* was used as the internal control. The data are presented as the mean ± S.D. values, *p < 0.05, **p < 0.01, Student’s *t* test.

The processes of cell motility and adhesion involve the extension and arrangement of cytoskeleton. The regulation of actin cytoskeleton pathway was also dominantly enriched by RASSF1A expression (Fig. S3). Although the final cell morphologies were not identical, the cells were subsequently subjected to F-actin staining. We observed a strikingly anchor-like pattern of F-actin in RASSF1A-overexpressing cells compared with control cells. In addition, more F-actin was arranged along the stretching direction in RASSF1A-depleted cells (Fig. 4a).

The transcriptional coactivator YAP1 has been reported to participate in EMT regulation and connect with RASSF1A signaling^10^. Nuclear localization of YAP1 is considered crucial for its activation. Nuclear YAP1 can bind to DNA-binding transcription factors (including TEAD) to regulate the expression of target genes. Activation of nuclear YAP1 was significantly inhibited in RASSF1A-overexpressing CNE-2 cells, as shown by the results of nuclear/cytoplasmic fractionation assays (Fig. 4b) and the concomitant downregulation of CTGF and CYR61, two well-known YAP1 target genes (Fig. 4c). In addition, we observed induced YAP1 nuclear localization and upregulation of two YAP1 target genes in RASSF1A-depleted CNE-1 cells (Fig. 4b, c). Our findings suggest a role for RASSF1A in modulating actin arrangement and YAP1 activation during NPC development.

## RASSF1A inhibits the expression of PDGFB *via* YAP1 inactivation

**Fig. 5:**
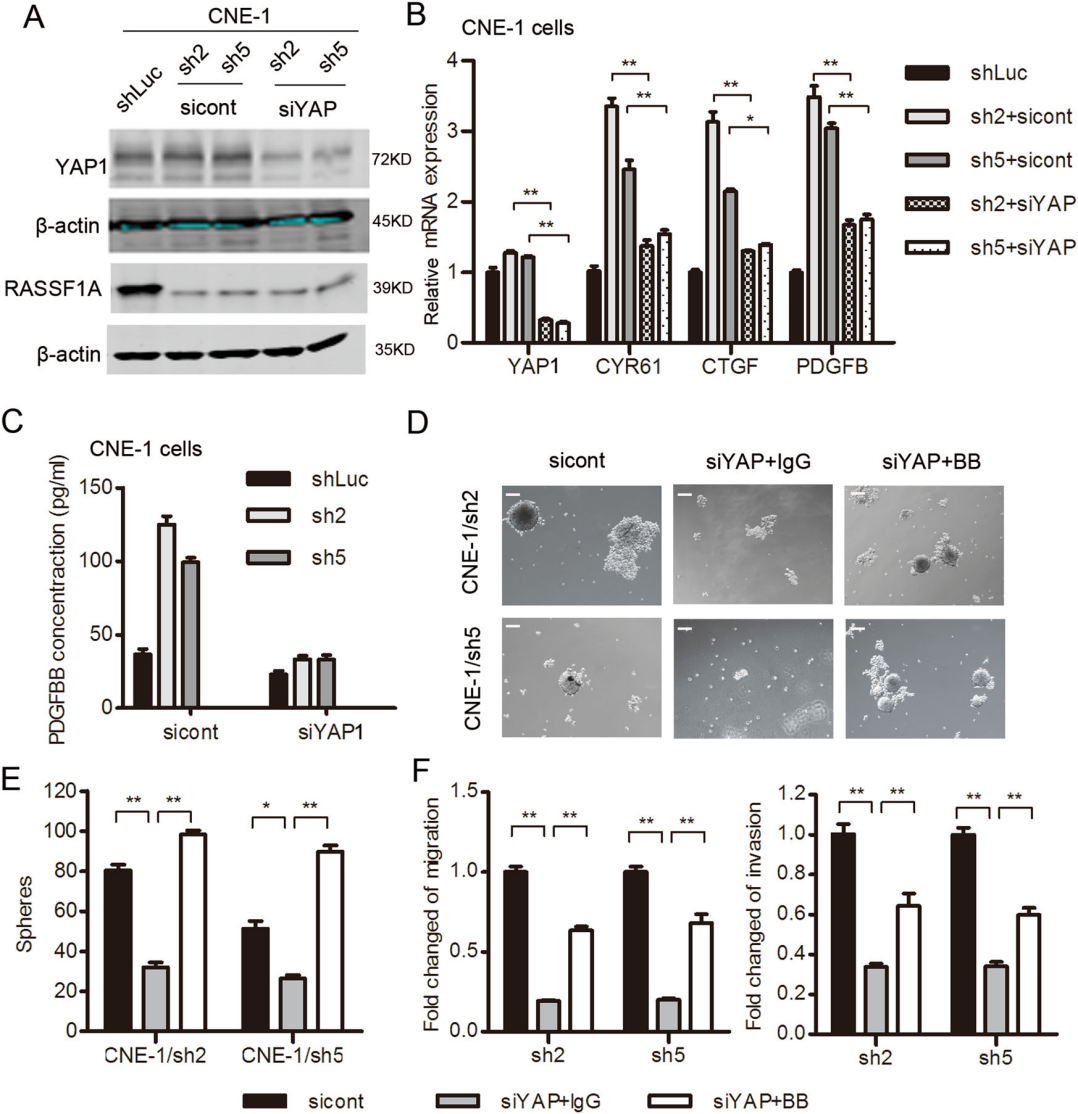
PDGFB induction by YAP1 mediates the modulatory effect of RASSF1A on the malignant phenotypes of NPC cells. **a**–**c** YAP1 was transiently knocked down in RASSF1A-depleted CNE-1 cells. **a** In the indicated cells, YAP1 protein expression was assessed by using western blotting; **b** *PDGFB, CYP61 and CTGF* mRNA expression was assessed by qRT-PCR; **c** Concentration of PDGF-BB secreted in CM was measured by ELISA. The data are presented as the mean ± S.D. values, *p < 0.05, **p < 0.01, Student’s *t* test. **d**–**f** Recombinant PDGF-BB or IgG was added to YAP1-silenced NPC cells. **d** The formed spheroids were counted via microscopy, and (**e**) representative images are shown. **f** The impact of PDGF-BB treatment on the migration and invasion of RASSF1A-depleted cells was determined by Transwell assays. The data are presented as the mean ± S.D. values, *p < 0.05, **p < 0.01, Student’s *t* test. Scale bar: 200 µm.

YAP1 has been confirmed to trigger *PDGFB* transcription by recruiting TEAD1^31^. To further investigate the involvement of YAP1 in linking RASSF1A with PDGFB, we transiently knocked down *YAP1* by using a pool of mixed siRNA in RASSF1A-depleted NPC cells, as confirmed by western blot analysis (Fig. 5a), and downregulated *CTGF* and *CYR61*, a hallmark of YAP1 activation as confirmed by qRT-PCR (Fig. 5b). Reduced expression of *PDGFB* and secretion of PDGF-BB in conditioned medium (CM) were found in YAP1-silenced RASSF1A-depleted CNE-1 cells compared with control RASSF1A-depleted CNE-1 cells (Fig. 5b, c) suggesting that YAP1 mediated the regulatory effect of RASSF1A on *PDGFB* expression. However, we did not observe any change in actin cytoskeletal rearrangement after YAP1 silencing (Fig. S4), indicating that cytoskeletal rearrangement might be upstream of YAP1 activation. To rule out the off-target of mixed siRNA mentioned above, we repeated the experiments with a single siRNA that has been reported^10^. We also observed transient knockdown of *YAP1*reduced PDGF-BB secretion and sphere formation (Fig. S5). Furthermore, human recombinant PDGF-BB protein was added to YAP1-silenced RASSF1A-depleted CNE-1 cells and resulted in increased sphere formation (Figs. 5d, e) and also increased migratory and invasive abilities (Fig. 5f) compared with those of IgG-treated RASSF1A-depleted CNE-1 cells. Long time of PDGF-BB treatment led to a spheroid-forming ability even higher than that of YAP1-expressing cells, possibly because of a autocrine PDGF-BB/PDGFR positive feedback loop^31^. Thus, RASSF1A mediated PDGFB inhibition *via* YAP1 inactivation in NPC cells.

### RASSF1A inactivates YAP1 through actin cytoskeletal rearrangement

**Fig. 6:**
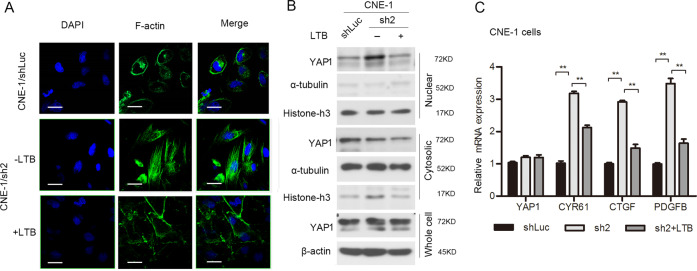
RASSF1A inactivates YAP1 through actin rearrangement. **a**–**c** RASSF1A-depleted CNE-1 cells were treated with or without LTB (10 µM) treatment for 1 h, and its control CNE-1 cells were also included into assays as follows. **a** Representative images of F-actin stained with phalloidin were observed by immunofluorescence microscopy. Nuclei were visualized by DAPI staining, scale bar: 25 µm. **b** YAP1 expression in the total, cytosolic or nuclear fractions was determined by western blot analysis. **c** mRNA quantification of *YAP1*, *CYR61*, *CTGF* and *PDGFB* by using qRT-PCR; *GAPDH* was used as the internal control. The data are presented as the mean ± S.D. values, **p < 0.01, Student’s *t* test.

As indicated above, actin arrangement remained unchanged in response to YAP1 transient knockdown. In order to substantiate that actin remodeling plays a role in regulating YAP1 activation, we treated RASSF1A-delepted cells with latrunculin b (LTB), an agent that selectively binds G actin and blocks the formation of F-actin (Fig. 6a) and found LTB treatment inhibited YAP1 nuclear localization (Fig. 6b) and prevented YAP1 -regulated gene (*CTGF* and *CYR61*) transcription (Fig. 6c) induced by RASSF1A silencing. Reduced expression of *PDGFB* (Fig. 6c) was also observed in LTB-treated RASSF1A-depleted CNE-1 cells, indicating that actin cytoskeletal rearrangement acts upstream of YAP1 nuclear import and subsequent activation in RASSF1A-modulated NPC cells.

## Discussion

The release of pro- and anti-inflammatory cytokines has a significant role in the triggering of malignant transformation and display of stem cell properties^32–34^ Gene profiling has yielded important mechanistic insights into the role of PDGFB signaling in RASSF1A-mediated stem cell plasticity. The PDGFB protein encoded by the *PDGFB* gene in humans is a member of the platelet-derived growth factor family that can be released into the microenvironment as a homodimer (PDGF-BB) or as a heterodimer with the platelet-derived growth factor alpha (PDGFA) polypeptide (PDGF-AB). Secreted PDGF-BB is involved in cancer progression *via* multiple mechanisms. PDGF-BB induces the stem cell phenotype either by accelerating the maturation of collagen chains through increased LOX activity^35^ or by inducing both perivascular and satellite cell gene expression to acquire improved migration of human hematopoietic cells^36,37^. Our data showed that *PDGFB* silencing abrogated the RASSF1A depletion-induced malignant phenotypes of NPC cells. Based on these results, *PDGFB* or PDGFBB may exhibit a developmental interest of a new therapeutic target in patients with late-stage malignant NPC.

*RASSF1A* acts as a natural barrier of stem cell self-renewal by allowing the quaternary association of YAP1-TEAD with the Oct4 enhancer^38^. Epigenetic silencing of RASSF1A results in constitutive nuclear YAP11 accumulation, which increases the extracellular matrix deposition and enhances stem-like characteristics^39^. Consistent with previous studies^40,41^, our study confirmed that RASSF1A depletion induced YAP1 nuclear translocation and triggered the expression of its target genes. Nuclear YAP1 is believed to activate TEAD transcriptional activity and induce the expression of a broad range of cytokines^42^. The level of YAP1 in human liver tissues is positively correlated with the expression of pro-inflammatory cytokines (including MCP-1, TNF-α and IL-6)^43^. Enhanced secretion of IL-6 by YAP1-activated hepatocellular carcinoma cells might induce tumor-associated macrophage maturation, and disruption of YAP1 function could suppress macrophage chemotaxis and migration^44^. Recently, YAP1 was reported to trigger *PDGFB* transcription by recruiting TEAD1 in bladder cancer cells, as *PDGFB* has a TEAD-binding motif in the gene promoter^31^. *PDGFB* expression was enhanced after over-expression of YAP1, whereas activated YAP1 downregulated the expression of IL-1α/β, consistent with our findings (data not shown)^45^. We also performed a rescue assay in RASSF1A-depleted NPC cells with transient knockdown of YAP1 and demonstrated that ectopic PDGF-BB treatment restored the inhibition of YAP1 knockdown-mediated malignant phenotypes, confirming that PDGFB is directly downstream of the RASSF1A/YAP1 axis.

YAP1 is regulated by mechanical cues *via* the interaction of Hippo pathway components with the cytoskeleton. Cell detachment activates Lats1/2 and leads to YAP1 inhibition through cytoskeletal reorganization, whereas detachment-induced YAP1 inactivation is required for anoikis in nontransformed cells^46^. The small GTPase Rho controls YAP1 nuclear localization by promoting the formation of actin bundles and stress fibers^47^. Simultaneously, YAP1 activation also creates a positive feedback loop to control cytoskeletal remodeling, in turn controlling EMT and cell metastasis. YAP1 activates the transcription of ARHGAP29 to suppress RhoA activity, resulting in metastasis promotion^48^. Our study analysis showed RASSF1A triggered F-actin rearrangement and subsequent YAP1 nuclear translocation. Knockdown of YAP1 had little effect on F-actin rearrangement, suggesting that actin remodeling acts as an upstream regulator of YAP1 activity in RASSF1A-mediated biological functions in NPC cells.

Polymerization and depolymerization of actin filaments is a dynamic process controlled by various regulatory proteins, including Rho family GTPases^49^. RASSF1A localizes mainly in the cytoplasm but can be recruited to the plasma membrane by activated RAS^1^. Ras signaling stimulates pathways toward the Rho GTPase family (RhoA/B/C, Rac, and Cdc42) activation. Indeed, Rac1 activation is increased in RASSF1A-knockdown cells^50^, and direct interaction of RASSF1A with RhoA suppresses the transforming activity of RhoA^51^. However we did not observed change of activity of Rac1 or RhoA in our cell model. Dubois et al. has reported RASSF1A controls PP2A/GEFH1/RhoB regulation of cofilin-regulated F-actin polymerization^10^. The mechanism by which RASSF1A triggers actin remodeling process in NPC cells needs further investigation.

In conclusion, the present study indicates that downregulation of RASSF1A is extensively involved in the acquisition of stem-like characteristics and the motility and invasive phenotypes of NPC cells. Mechanistically, RASSF1A leads to YAP11 inactivation by remodeling F-actin assembly and subsequently inhibits the transcriptional activity of PDGFB, an important target required for sustaining the malignant phenotypes of NPC cells.

## Supplementary information

supplementary materials

supplementary figure legends

fig.s1

fig.s2

fig.s3

fig.s4

fig.s5
