# Supplementary material for: RASSF1A inhibits PDGFB-driven malignant phenotypes of nasopharyngeal carcinoma cells in a YAP1-dependent manner
Source: Cell Death Dis. 2020 Oct 14;11(10):855. doi: 10.1038/s41419-020-03054-z (PMC7560678; doi:10.1038/s41419-020-03054-z)
Supplement: Supplementary file 1 — supplementary materials [file 41419_2020_3054_MOESM1_ESM.docx]

**Supplementary Materials & Methods**

The sequences of the PCR amplification primers were as follows: *GAPDH* forward, 5’-AAGGTCATCCCTGAGCTGAA-3’;*GAPDH* reverse, 5’-TGACAAAGTG GTCGTTGAGG-3’; *YAP1* forward, 5’-TGACCCTCGTTTTGCCATGA-3’,*YAP1* reverse, 5’-GTTGCTGCTGGTTGGAGTTG-3’; *PDGFB* forward,5’-GCTCTTCCTGTCTCTCTGCTG-3’, *PDGFB* reverse, 5’-AGATTGGCTTCTTCCGCACA-3’;*CYR61* forward, 5’-CAGGACTGTGAAGATGCGGT-3’, *CYR61* reverse, 5’-AGCCTGTAGAAGGGAAACGC-3’; *CTGF* forward, 5’-CACCCGGGTTACCAATGACA-3’; and *CTGF* reverse, 5’-TCCGGGACAGTTGTAATGGC-3’. *RASSF1A* forward, 5'- GCGCGCATTGCAAGTTCA-3', RASSF1A reverse, 5'- TTGGATCTTCTGGCGGCAAT-3'; *RASSF1B* forward, 5’-CCCAGGTGGCCAACATTAGA-3’, *RASSF1B* reverse,5’- CAGCATCCTTGGGCAGGTAA-3’;*RASSF1C* forward, 5′-TACTGCAGCCAAGAGGACTCGG-3′, *RASSF1C* reverse, 5′-TCAGGTGTCTCCCACTCCACAG-3′; *RASSF1D* forward, 5’-CCTCTCTGCAGATTGCAAGTTC-3’, *RASSF1D* reverse, 5’-CAGCATCCTTGGGCAGGTAA-3′.
