# Supplementary material for: RASSF1A inhibits PDGFB-driven malignant phenotypes of nasopharyngeal carcinoma cells in a YAP1-dependent manner
Source: Cell Death Dis. 2020 Oct 14;11(10):855. doi: 10.1038/s41419-020-03054-z (PMC7560678; doi:10.1038/s41419-020-03054-z)
Supplement: Supplementary file 2 — supplementary figure legends [file 41419_2020_3054_MOESM2_ESM.docx]

Fig.S1 Image of tumors formed in mice.

(Upper) Tumors formed in mice implanted with RASSF1A-overexpressing and empty vector-expressing CNE-2 cells and their control cells. (Lower) Tumors formed in mice implanted with shRNA-mediated RASSF1A knockdown CNE-1 cells. Scale bar: 1cm.

Fig.S2 Restoration of RASSF1A reduced the ability of proliferation and sphere formation in RASSF1A knockdown CNE-1 cells.

(A, B, C) RASSF1A knockdown CNE-1 cells stably transfected with silent mutated of RASSF1A-expressing or empty(contral) vector were analyzedas follows. (A)Protein expression levels of RASSF1A were determined by western blot analysis; GAPDH was used as the loading control. (B) A cell proliferation curve was constructed from MTS assay results, the data are presented as the mean± S.D. values, ***p* < 0.01 for comparison between CNE-1/sh2 cells transfected with RASSF1A or vector, ^##^ *p* < 0.01 for comparison between CNE-1/sh5 cells transfected with RASSF1A or vector; Student’s t-test. (C) Single-cell suspensions were seeded and the formed spheroids were counted *via* microscopy, and representative images are shown. The representative images and numbers were compared, ***p* < 0.01, Student’s t-test. Scale bar: 1mm. (D) mRNA expression of RASSF1 isoforms in indicated cells. (E) Cell proliferation was measured by MTS absorbance in CNE1 cells with RASSF1B or RASSF1C re-expression. (F) Single-cell suspensions were seeded and the formed spheroids were counted *via* microscopy, and the numbers were compared. Student’s t-test. ns: not significant.

Fig.S3 Pathways enriched in RASSF1A-overexpressing CNE-2 cells and RASSF1A-depleted CEN-1 cells compared with their corresponding control cells.

Fig.S4 Knockdown of *YAP*1 does not affect actin remodeling in RASSF1A-delepted NPC cells.

Representative images of F-actin stained with phalloidin. Nuclei were visualized by DAPI staining. Scale bar: 25µm.

Figure S5 The effects of transient silencing of *YAP1* by another single siRNA against *YAP1*

(A) *PDGFB, CYP61, CTGF and PDGFB* mRNA expression was assessed by qRT-PCR; (B) Concentration of PDGF-BB secreted in CM was measured by ELISA. The data are presented as the mean± S.D. values, **p* < 0.05, ***p* < 0.01, Student’s t-test. (C) The formed spheroids were counted via microscopy, and (left) representative images are shown. The numbers (right) were compared, ***p* < 0.01, Student’s t-test. Scale bar: 1mm.
