# Supplementary figures and images for: RASSF1A inhibits PDGFB-driven malignant phenotypes of nasopharyngeal carcinoma cells in a YAP1-dependent manner
Source: Cell Death Dis. 2020 Oct 14;11(10):855. doi: 10.1038/s41419-020-03054-z (PMC7560678; doi:10.1038/s41419-020-03054-z)

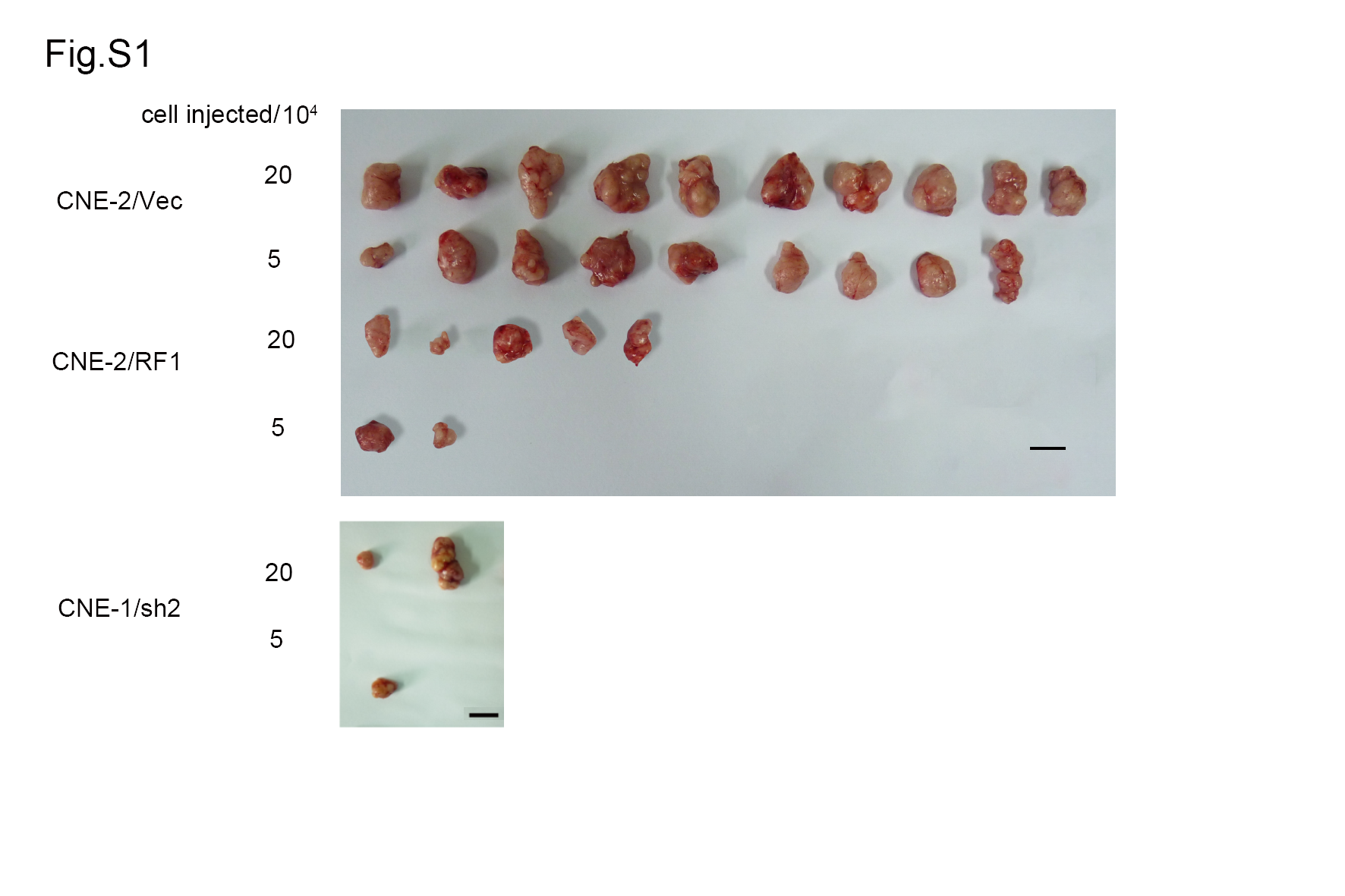

Supplement: Supplementary file 3 — fig.s1 [file 41419_2020_3054_MOESM3_ESM.tif]

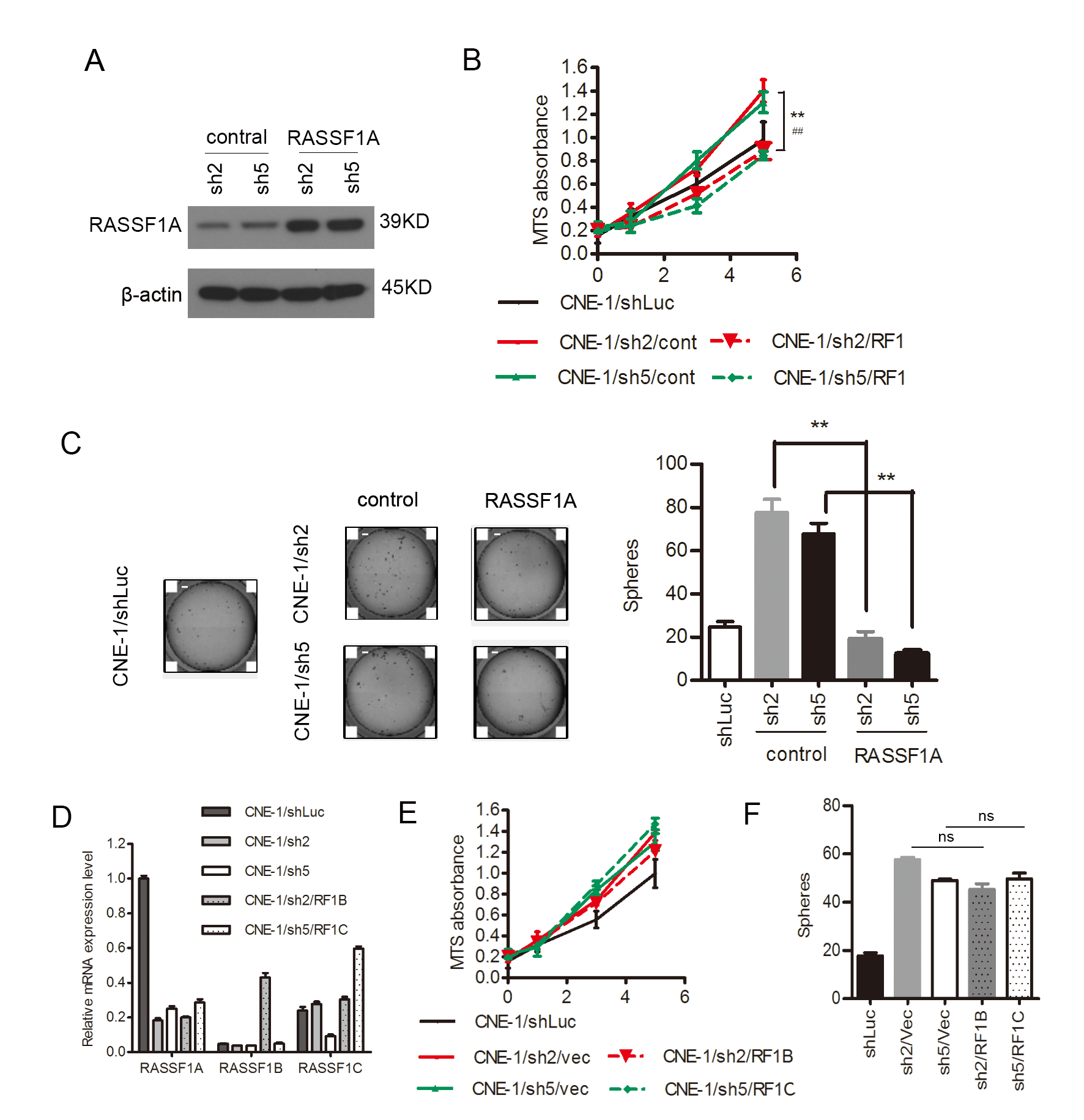

Supplement: Supplementary file 4 — fig.s2 [file 41419_2020_3054_MOESM4_ESM.tif]

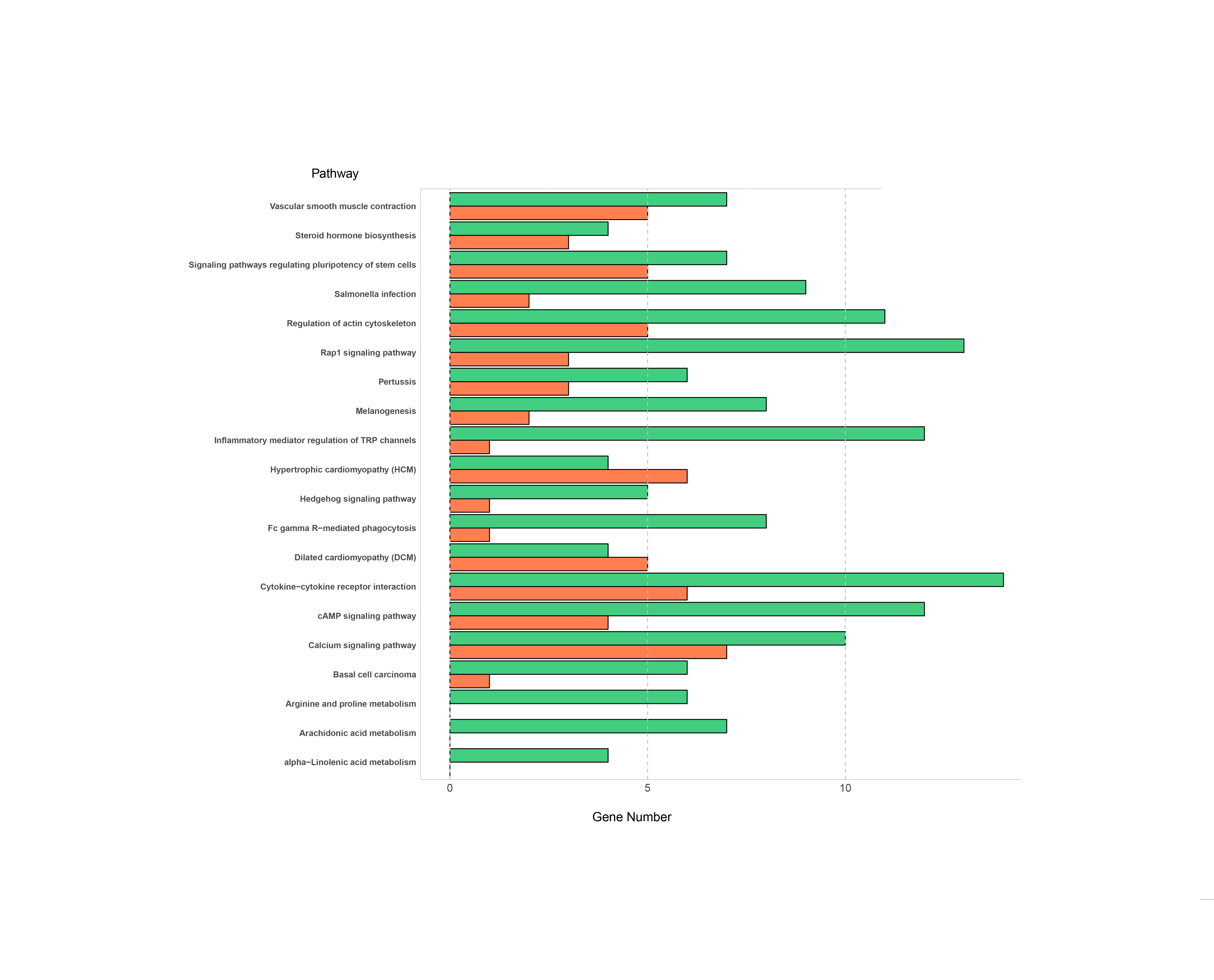

Supplement: Supplementary file 5 — fig.s3 [file 41419_2020_3054_MOESM5_ESM.tif]

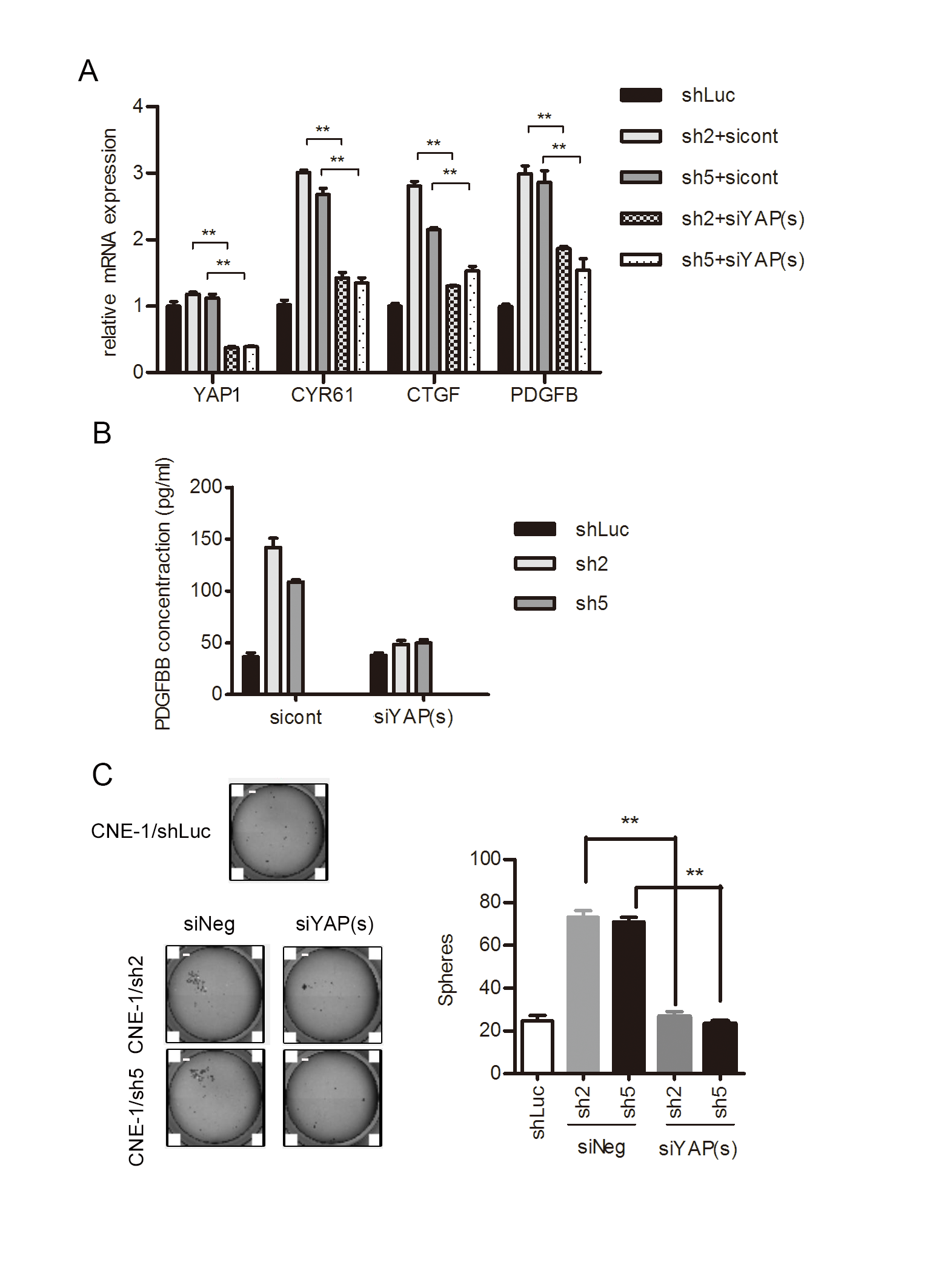

Supplement: Supplementary file 7 — fig.s5 [file 41419_2020_3054_MOESM7_ESM.tif]
